# Use of transcutaneous spinal cord stimulation to explore inhibitory and facilitatory circuits in muscles of the human lower limb

**DOI:** 10.1113/EP093023

**Published:** 2025-07-21

**Authors:** Julia Sordet, Maria Papaiordanidou, Nicolas Amiez, Ioannis Amiridis, Jean‐Pierre Quenot, Alain Martin

**Affiliations:** ^1^ Université Bourgogne Europe, Inserm CAPS UMR 1093 Dijon France; ^2^ Laboratoire Interuniversitaire de Biologie de la Motricité (LIBM), Univ Lyon Université Claude Bernard Lyon 1 Villeurbanne France; ^3^ Department of Physical Education and Sport Sciences at Serres Aristotle University of Thessaloniki Thessaloniki Greece; ^4^ Service de Médecine Intensive‐Réanimation CHU Dijon‐Bourgogne Dijon France

**Keywords:** heteronymous facilitation, H reflex, posterior root muscle reflex, presynaptic inhibition, primary afferent depolarization

## Abstract

The aim of this study was to explore the primary afferent depolarization mechanism, to determine whether the soleus transspinal evoked potential (TEP), elicited through transcutaneous spinal cord stimulation over the L1–L2 level, is modulated by presynaptic inhibition and heteronymous facilitation, similar to the Hoffmann (H) reflex, elicited by posterior tibial nerve stimulation. Twenty subjects participated in two experiments. Experiment 1 assessed D_1_ and D_2_ inhibition by conditioning the H reflex and TEP with peroneal nerve stimulation at different interstimulus intervals (ISIs; ranging from 1 to 200 ms). Experiment 2 examined heteronymous facilitation of responses using femoral nerve conditioning stimulation (ISIs ranging from −1 to −10 ms). Conditioned responses (H_PSI_ or TEP_PSI_ and H_FAC_ or TEP_FAC_) were compared with unconditioned ones (H_TEST_ or TEP_TEST_). Concerning D_1_ and D_2_ inhibition, results did not reveal any significant difference between the two responses (*p *= 0.89 and *p *= 0.51 for D_1_ and D_2_, respectively). Inhibition was observed at all ISIs for D_1_ and at the 100 and 200 ms ISIs for D_2_. Facilitation patterns were also comparable between the two responses. Moreover, a negative correlation was observed between the modulation of soleus TEP and tibialis anterior TEP (conditioning muscle during inhibition), whereas a positive correlation was obtained between soleus TEP and quadriceps TEP (conditioning muscle during facilitation). The similar modulations between the two responses suggest that TEP can be an alternative to the H reflex for studying spinal circuits, with the advantage of offering insight into the activity of multiple lower‐limb muscles.

## Abstract (highlights)

- **What is the central question of this study?**
  Is the transspinal evoked potential, elicited by transcutaneous spinal cord stimulation, sensitive to inhibitory and facilitatory circuits, similar to the H reflex evoked by peripheral nerve stimulation?
- **What is the main finding and its importance?**
  Both soleus H reflex and transspinal evoked potential are sensitive to D_1_ and D_2_ inhibition, mediated by activation of the antagonist tibialis anterior afferents, and to heteronymous facilitation, induced by projections of vastus lateralis afferents. These findings suggest that transcutaneous spinal cord stimulation is a valuable tool for investigating spinal circuits, offering the advantage of assessing multiple lower‐limb muscles simultaneously.

## 1 INTRODUCTION

Transcutaneous spinal cord stimulation (tSCS) is a non‐invasive technique that is increasingly being used in clinical practice, particularly for patients with neurological disorders (Hofstoetter & Minassian, 2022; Hofstoetter et al., 2009, 2013; Megía García et al., 2020; Minassian et al., 2024). This method involves the transcutaneous delivery of electrical current through electrodes placed along the spinal cord. When positioned at the lumbar or thoracic levels, these electrodes induce simultaneous responses across multiple muscles of the lower limbs (Maertens De Noordhout et al., 1988), referred to as transspinal evoked potentials (TEPs; Knikou, 2015, 2013, 2017). These responses are driven primarily by the activation of large‐diameter somatosensory afferents located close to the paravertebral electrodes. Research has shown that TEPs are depressed during paired‐pulse stimulations (Andrews et al., 2015; Hofstoetter et al., 2018; Minassian et al., 2007) or during the application of local vibration (Courtine et al., 2007; Gravholt et al., 2024; Minassian et al., 2007), reflecting the characteristic behaviour of activation of afferent fibres and, therefore, the reflex nature of the response.

In humans, non‐invasive activation of afferent fibres is generally achieved by peripheral nerve stimulation. A low stimulation intensity applied over a peripheral nerve evokes a monosynaptic reflex named the Hoffmann reflex (H reflex; Zehr, 2002). This reflex is widely used to explore the neural circuitry of the human spinal cord and serves as a valuable tool for assessing the effectiveness of activated Ia afferents in generating action potentials in motoneurons (Schieppati, 1987; Theodosiadou et al., 2023). However, obtaining the H reflex can be challenging in certain muscle groups (e.g., quadriceps) (Doguet & Jubeau, 2014), particularly in resting conditions, owing to the technical difficulties involved in stimulating Ia afferents (Zehr, 2002). As a result, when focusing on the lower limb, the posterior tibial nerve is often targeted for stimulation, because the H reflex can be evoked easily in the soleus muscle, even at rest.

Although both tSCS and peripheral nerve stimulation can elicit reflex responses through afferent solicitation, the nature of the tSCS responses appears far more complex than the H reflex. In addition to afferent recruitment, tSCS can also activate motor axons (Roy et al., 2012) and even the corticospinal tract (Škarabot et al., 2019), depending on the stimulation parameters (Binder et al., 2021; Danner et al., 2016; Krenn et al., 2023, 1996; Sayenko et al., 2015; Skiadopoulos et al., 2022). Peripheral nerve stimulation generates two distinct responses in the EMG signal [a direct response stemming from activation of motor axons (the M‐wave) and an indirect response from Ia afferent depolarization (the H reflex)], whereas tSCS produces a single composite response, reflecting the simultaneous activation of both afferent and efferent pathways (Minassian et al., 2007). It is noted that the sensory and motor contributions to the TEP are influenced by various stimulation parameters, such as electrode placement, intensity of stimulation, spine curvature or body position (Binder et al., 2021; Danner et al., 2016; Krenn et al., 2015; Roy et al., 2012). Additionally, unlike the H reflex, which is mediated primarily by homonymous afferent activation, tSCS‐evoked responses can be modulated by the interaction of synergist or antagonist muscles [e.g., reciprocal inhibition, heteronymous facilitation (HF)], owing to the simultaneous activation of multiple muscles (Minassian et al., 2007).

Despite these differences, the significant advantage in evoking responses simultaneously across multiple muscles has positioned tSCS as a promising tool for evaluating the spinal circuitry (Nakagawa et al., 2024; Saito et al., 2019), offering an alternative to the H reflex. The study of spinal circuitry relies on afferent activation that can be modulated at rest by presynaptic inhibitory mechanisms. One of the most studied mechanisms is primary afferent depolarization (PAD), which results in reduced neurotransmitter release at the synapse between Ia afferents and α‐motoneurons (Crone & Nielsen, 1989; Rudomin & Schmidt, 1999). This depolarization is mediated primarily by inhibitory interneurons activated by the afferents of the antagonist (Hari et al., 2022; Metz et al., 2023). Primary afferent depolarization has been studied extensively using the H reflex and is typically assessed through methods such as D_1_ or D_2_ presynaptic inhibition (refer to early and late phases of inhibition, respectively) and HF (Pierrot‐Deseilligny & Burke, 2005). However, its modulation through tSCS remains unexplored. Investigating TEP modulation through both inhibitory and facilitatory spinal circuits could provide valuable insights into the nature of tSCS‐induced responses and support its use as an alternative method for assessing the effectiveness of Ia afferent activation in eliciting motoneuron responses.

Therefore, the aim of the present study was to compare the influence of D_1_ and D_2_ presynaptic inhibition and HF on the soleus H reflex and TEP, to investigate whether the two responses are influenced in the same way by these inhibitory and facilitatory spinal circuits. Additionally, the multi‐segmental responses evoked by tSCS allowed for an investigation of the modulation of multiple lower‐limb muscles during these inhibitory and facilitatory manoeuvres. In stimulation conditions that promote a similar afferent contribution between H reflex and TEP of the soleus muscle, it was hypothesized that the modulations would be similar between these two responses and that these modulations would also extend to synergistic and antagonistic muscles.

## 2 MATERIALS AND METHODS

### 2.1 Participants

Twenty healthy participants (10 females, 27.3 ± 8.97 years, 66.05 ± 13.83 kg and 174 ± 9.84 cm) with no history of neuromuscular disorders volunteered to participate in this study. After being informed about the objectives of the study and the potential adverse effects associated with their participation, they all gave their written informed consent. The experimental design was approved by the local ethics committee (ID: 171812) and was in accordance with the principles of the *Declaration of Helsinki* for human experimentation, except for registration in a database.

### 2.2 Study design

**FIGURE 1:**
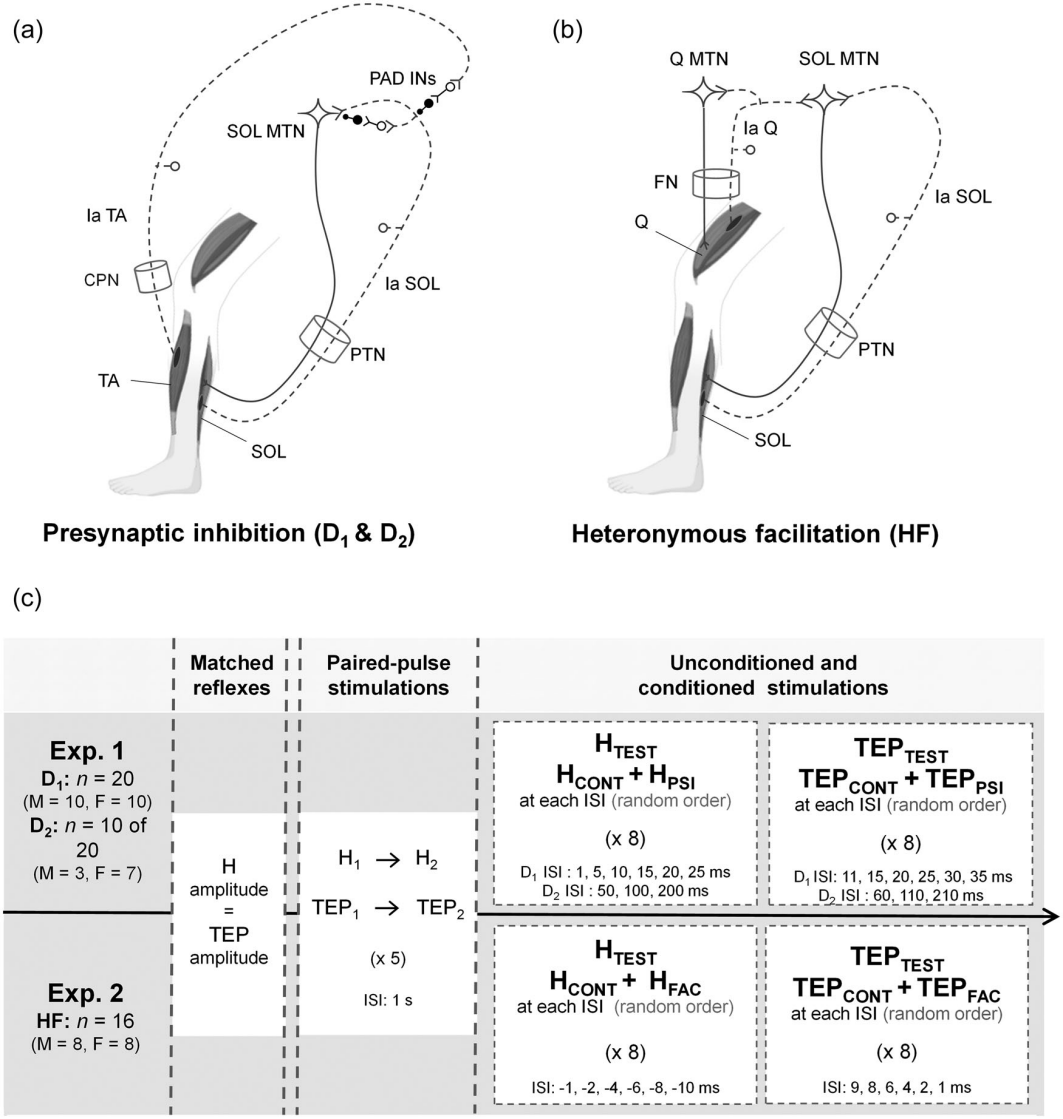
Experimental design. (a) Representation of neural pathways inducing D_1_ and D_2_ inhibition (according to Metz et al., 2023). A prior conditioning stimulation applied to the common peroneal nerve (CPN) induces a decrease of the SOL Hoffmann (H0 reflex (conditioned response) compared with the unconditioned response. Stimulation of TA Ia afferents activates PAD Ins, which project on SOL Ia afferents. Abbreviations: Ia, Ia afferents; MTN, motoneuron; PAD INs, PAD interneurons; PTN, posterior tibial nerve; SOL, soleus, TA, tibialis anterior. (b) Representation of neural pathways inducing heteronymous facilitation (HF) (according to Pierrot‐Deseilligny & Burke, 2005). A prior stimulation applied to the femoral nerve increases the amplitude of the H reflex (conditioned response) compared with the unconditioned response. Quadriceps Ia afferents have facilitatory monosynaptic projections on SOL MTN. Abbreviations: FN, femoral nerve; Q, quadriceps. (c) Study design. Twenty volunteers (*n *= 20) were included in this study. All of them participated in the first experiment (Experiment 1) to evaluate D_1_ presynaptic inhibition (D_1_). For 10 of them, D_2_ presynaptic inhibition (D_2_) was also tested during this experiment. Sixteen individuals took part in the second experiment (Experiment 2), conducted to evaluate heteronymous facilitation. After matching the amplitude of the H reflex and TEP, paired‐pulse stimulations were delivered at 1 s ISI. For both experiments, test stimulations were delivered first, then conditioned stimulations at each ISI, and a series of control stimulations were delivered in a random order. Abbreviations: Exp. 1, Experiment 1; Exp. 2, Experiment 2; F, female; H_CONT_ and TEP_CONT_, control series of unconditioned responses obtained during the stimulation protocol; HF, heteronymous facilitation; H_FAC_ and TEP_FAC_, series of conditioned responses during HF; H_PSI_ and TEP_PSI_, series of conditioned responses during D_1_ and D_2_ inhibition; H_TEST_ and TEP_TEST_, first series of unconditioned responses obtained at the beginning of the stimulation protocol; H_1_ and TEP_1_, first response of paired‐pulse stimulations; H_2_ and TEP_2_, second response of paired‐pulse stimulations; ISI, interstimulus interval (a negative ISI indicates that the test stimulation was delivered before the conditioning stimulation); M, male; TEP, transspinal evoked potential.

To test D_1_/D_2_ presynaptic inhibition (Figure 1a) and HF (Figure 1b), participants visited the laboratory on two occasions to take part in two distinct experimental sessions, each lasting ∼90 min. These sessions were separated by a minimum of 48 h and were carried out randomly. The first experiment (Experiment 1, *n* = 20) was conducted to examine D_1_ inhibition (for 10 of the 20 participants, D_2_ inhibition was also tested). The second experiment (Experiment 2) aimed at evaluating HF. As previously reported (Bergmans et al., 1978; Grosprêtre et al., 2019), it was not possible to obtain HF on every participant. In the present study, HF was obtained in only 16 of the 20 initial participants. During the two experimental sessions, posterior tibial nerve stimulation (PTNS) and tSCS were delivered to evoke soleus (SOL) H reflex and TEP, respectively. Stimulation parameters for PTNS and tSCS were determined to evoke an equal amplitude of H reflex (H_TEST_) and TEP (TEP_TEST_) of the right SOL muscle, which was the target muscle. To ensure that both PTNS and tSCS stimulation activated afferent pathways, the post‐activation depression phenomenon was tested in the initial phase of each experimental session. Then, the intensities of the conditioning stimulations, common peroneal nerve for Experiment 1 and femoral nerve for Experiment 2, were identified. Subsequently, SOL unconditioned H reflexes (H_TEST_) and TEPs (TEP_TEST_) of matched amplitude were delivered. Finally, conditioned H reflexes (H_PSI_ for inhibition or H_FAC_ for facilitation) and conditioned TEPs (TEP_PSI_ or TEP_FAC_) at different interstimulus intervals (ISIs) and control unconditioned H (H_CONT_) and TEPs (TEP_CONT_) were delivered in a random order. It is important to note, however, that a temporal separation (∼5 min) between test and control stimulations was applied to verify the consistency of the test reflex amplitude throughout the sessions. The experimental design is presented in Figure 1c.

### 2.3 Participant position

Participants were in a supine position on an isokinetic dynamometer (Biodex Medical Systems, SYSTEM 4, Inc., Shirley, NY, USA), favouring activation of sensory fibres during tSCS (Danner et al., 2016). The right foot was strapped to the pedal of the dynamometer at the ankle level, with the ankle joint at 90° and the knee and hip joints at 0° (full extension). The left foot was installed on a table at the same height as the right foot.

### 2.4 EMG recordings

After shaving and dry‐cleaning the skin with alcohol to obtain low impedance (<5 kΩ), two silver chloride circular (7 mm recording diameter) surface electrodes for EMG recording (Contrôle Graphique Medical, Brie‐Compte‐Robert, France), with an interelectrode centre‐to‐centre distance of 2 cm, were positioned over the soleus (SOL), gastrocnemius lateralis (GL), gastrocnemius medialis (GM), tibialis anterior (TA) and vastus lateralis (VL) muscles of the right leg and on SOL (L SOL) and VL (L VL) of the left leg. For left and right SOL, electrodes were positioned 2 cm below the insertion of the two gastrocnemii over the Achilles tendon. For GL, GM and right and left VL muscles, electrodes were placed over the muscle belly, and for TA the electrodes were positioned at the upper third of the distance between the fibular head and the tip of the lateral malleolus. The common reference electrode was placed between upper gastrocnemii insertions of the right leg. EMG signals were amplified (gain = 1000), filtered (1 Hz–5 kHz), and collected with a sampling frequency of 1000 Hz using the BIOPAC System (Biopac MP150, Biopac System, Inc.).

### 2.5 Common experimental procedures

The procedures described hereafter were common between the two experimental sessions and were obtained in resting conditions. The H reflexes of SOL were elicited by stimulating the posterior tibial nerve in the popliteal fossa. A stimulator (DS7AR, Digitimer, Welwyn Garden City, UK) was used to deliver monophasic rectangular pulses (1 ms duration). A self‐adhesive anode (5 cm × 10 cm; Compex SA, Ecublens, Switzerland) was placed over the patella. The optimal cathode placement (i.e., the stimulation site permitting the highest electrophysiological SOL response to be obtained for the lowest stimulation intensity) was identified initially using a hand‐held cathode ball electrode (0.5 cm in diameter), which was then replaced by an electrode (30 mm in diameter; Durastick Premium, DJO Global, Vista, CA, USA) that was fastened securely at the popliteal fossa using straps. To determine H_TEST_ amplitude, the H reflex recruitment curve was built. To that end, three stimulations were delivered at each intensity every 10 s, starting from H reflex threshold (i.e., the lowest intensity giving the smallest response) until the maximal response amplitude (H_max_), with 2 mA increments. The intensity permitting an H reflex of ∼80% H_max_ (H_TEST_) to be evoked was then identified (27.22 ± 11.19 mA corresponding to 78.76 ± 13.14% H_max_ and 25.5 ± 16.40 mA corresponding to 80.94 ± 10.29% H_max_ for Experiments 1 and 2, respectively). After that, stimulations were delivered by increasing the intensity by increments of 10 mA until the evoked response reached a plateau (M_max_). This intensity was increased by a further 30%, to ensure supramaximal stimulation (102.65 ± 28.46 and 96.29 ± 39.22 mA for Experiments 1 and 2, respectively), and three M_max_ obtained at this intensity were recorded.

The tSCS‐evoked responses were evoked on muscles of the lower limb by means of tSCS. Stimulation was delivered using a stimulator (DS7AR, Digitimer), delivering monophasic pulses of 1 ms duration. The anode (5 cm × 10 cm; Durastick Plus, DJO Global, Vista, CA, USA) was placed longitudinally over the abdomen, under the umbilicus. The cathode (5 cm × 10 cm; Durastick Plus, DJO Global) was placed longitudinally over the paravertebral region, with its centre aligned between the spinous processes of the L1 and L2 vertebrae and positioned along the midline, equidistant between the left and right paravertebral areas. This electrode placement was used because it has been shown to promote activation of sensory fibres of SOL muscle and activation of motor axons of VL (Roy et al., 2012), thereby avoiding heteronymous facilitation from VL onto the SOL TEP. To set SOL TEP_TEST_ intensity, tSCS stimulations were delivered with an increment of 5 mA (three stimulations per intensity delivered every 10 s) from TEP threshold until the amplitude corresponded to the one of H_TEST_ (71.55 ± 22.75 mA for Experiment 1 and 66.76 ± 28.53 mA for Experiment 2).

During each experimental session, a control series of test responses (H_CONT_ and TEP _CONT_) was delivered at a random time point to ensure the consistency of stimulation conditions throughout the protocol. Because the H reflex and TEP were variable between trials, eight stimulations were delivered in each series of unconditioned and conditioned reflexes (Koceja, 2002; Papitsa et al., 2022). Once the intensities of test responses were obtained, the post‐activation depression phenomenon was assessed, for both the H reflex and TEP, by delivering a series of five paired‐pulse stimulations for each type of stimulation with a 1 s interval between each stimulation.

### 2.6 Experiment 1

This first experiment aimed at examining the degree of D_1_ and D_2_ presynaptic inhibition on SOL H reflex and TEP. The D_1_ and D_2_ presynaptic inhibition was assessed by conditioning the SOL H reflex or TEP by a prior stimulation of the common peroneal nerve. When Ia afferents of the antagonist TA muscle are activated, the H reflex is decreased owing to post‐activation depression of the SOL Ia afferents, triggered by PAD‐evoked spikes and/or by a long‐lasting inhibition of the SOL motoneurons (Metz et al., 2023). This inhibition has two phases: the first phase, termed D_1_ inhibition, is present from 5 to 30 ms, and the second phase, called D_2_ inhibition, is present from 70 to 200 ms (El‐Tohamy & Sedgwick, 1983; Mizuno et al., 1971). The common peroneal nerve was stimulated via two silver chloride surface electrodes (7 mm in diameter), placed on the upper part of the anterolateral side of the leg, distal to the fibular head (Forget et al., 1989). The conditioning stimulation was a triplet at 300 Hz (1 ms pulse duration) delivered at an intensity corresponding to 1–1.2 × TA motor threshold, defined as the lowest stimulation intensity that allowed recording of a TA M‐wave (11.9 ± 8.66 mA). The conditioning stimulus was applied at various ISIs prior to the test stimulation. These ISIs were obtained considering the delay between the end of the conditioning stimulation (end of the third pulse) and the beginning of the test stimulation. After preparation and installation of the participants, the determination of the intensities for H_TEST_, TEP_TEST_ and M_max_ was performed, as previously described in the common experimental procedures. Data recordings included two series of eight unconditioned H‐reflex (H_TEST_, delivered at the beginning of the session and H_CONT_, delivered in a random order to control the constancy of the test reflex amplitude during the session), six series of eight conditioned H reflexes with prior stimulation of the common peroneal nerve (H_PSI_) evoked at different ISIs (1, 5, 10, 15, 20 and 25 ms), two series of eight unconditioned TEP (TEP_TEST_, delivered at the beginning of the session and TEP_CONT_, delivered in a random order to control the constancy of the test reflex amplitude during the session) and six series of eight conditioned TEP with prior stimulation of the common peroneal nerve (TEP_PSI_) evoked at different ISIs. Because the delay in the appearance of TEP (19.65 ± 1.42 ms) is shorter than the delay in appearance of the H reflex (29.5 ± 2.01 ms), 10 ms was added at each abovementioned interval for tSCS stimulation, giving the following ISIs: 11, 15, 20, 25, 30 and 35 ms between common peroneal nerve and tSCS stimulation. For the 10 subjects for whom D_2_ was also assessed, three additional ISIs were tested (50, 100 and 200 ms for PTNS; and 60, 110 and 210 for tSCS). The series of stimulations were delivered in a random order with 10 s rest between each stimulation and 1 min rest between each series. During the experiment, all measurements were inspected visually to ensure no inadvertent muscle activity during stimulation. All trials where muscle activity was detected were removed, and further trials were performed.

### 2.7 Experiment 2

The aim of the second experiment was to examine the degree of HF between PTNS and tSCS stimulation. The SOL HF was assessed by a conditioning stimulus applied to the femoral nerve. Prior activation of the femoral nerve facilitates SOL responses because of the monosynaptic projections of quadriceps afferents on SOL α‐motoneurons (Hultborn et al., 1987). An electrical stimulator (DS7AR, Digitimer), delivering electrical stimulations of 1 ms pulse duration, was used for femoral nerve stimulation. The anode (5 cm × 10 cm; Compex SA) was placed on the right gluteus maximus muscle belly. The femoral nerve was stimulated initially using a cathode ball electrode placed in the femoral triangle, laterally to the femoral artery. Once the stimulation site was determined, the cathode ball was replaced by an electrode (a self‐adhesive electrode 30 mm in diameter; Durastick Plus, DJO Global), which was securely strapped to the stimulation site. Single stimulations were delivered at an intensity corresponding to 1–1.2 × VL motor threshold (i.e., lowest stimulation intensity allowing a VL M‐wave to be recorded, 42.59 ± 19.51 mA).

After the abovementioned common procedures, data recordings included two series of eight unconditioned H reflexes (H_TEST_ and H_CONT_), six series of eight conditioned H reflexes with prior activation of the femoral nerve (H_FAC_) evoked at different ISIs, two series of eight unconditioned TEP (TEP_TEST_ and TEP_CONT_) and six series of eight conditioned TEP with prior activation of the femoral nerve (TEP_FAC_) evoked at different ISIs, delivered in a random order. Given that the femoral nerve stimulation site is closer to the spinal cord than the posterior tibial nerve, the conditioning femoral nerve stimulation was delivered after the PTNS at different ISIs: −1, −2, −4, −6, −8 and −10 ms. The ISI adjustment, owing to the shorter appearance delay of TEP compared with the H reflex, gave the following ISIs for tSCS: 9, 8, 6, 4, 2 and 1 ms, with the femoral nerve stimulation delivered before tSCS. It should be noted that the 1 ms ISI was used for tSCS because the external triggering of the stimulators did not permit the two stimulations to be delivered at the same time.

### 2.8 Data analysis

In the case of PTNS, analysis was performed exclusively on the right SOL muscle, which was the target muscle, whereas for tSCS, analysis was performed on all recorded muscles. Data of some participants were removed from the analysis owing to problems with the EMG signal (for Experiment 1, data of two participants for GM, one participant for R VL and four participants for L VL; and for Experiment 2, data of one participant for R VL and of three participants for L VL).

The presence of the post‐activation depression phenomenon, tested in the initial phase of each experiment, was evaluated by comparing the peak‐to‐peak amplitude of the first response (i.e., H_1_ or TEP_1_) and the second response (i.e., H_2_ or TEP_2_) of the paired‐pulse stimulations at 1 s interval. The H_2_/H_1_ and TEP_2_/TEP_1_ ratios were also calculated to assess the degree of inhibition associated with this mechanism.

The peak‐to‐peak amplitude of H_TEST_, H_CONT_, TEP_TEST_, TEP_CONT_, H_PSI_, TEP_PSI_, H_FAC_, TEP_FAC_ and M_max_ was measured. Moreover, for H_TEST_ and H_CONT_, the amplitude of M_at_H_TEST_ and M_at_H_CONT_ (i.e., the M‐wave accompanying the H reflex) was also measured. The mean of each series (test, control and conditioned responses for each ISI) was calculated. For SOL, all these measures were normalized with respect to the mean of the three M_max_, giving the following ratios: H_TEST_/M_max_, TEP_TEST_/M_max_, H_CONT_/M_max_, TEP_CONT_M_max_, H_PSI_/M_max_, TEP_PSI_/M_max_, H_FAC_/M_max_, TEP_FAC_/M_max_ and M_at_H_TEST_/M_max_.

For tSCS‐evoked responses of the other muscles, raw values (expressed in millivolts) were standardized using *z*‐scores relative to the test response for each participant and muscle. The *z*‐score was calculated as:

$$z=\frac{\left(\mathrm{TE}P_{\mathrm{CONDITION}}-\mu \mathrm{TE}P_{\mathrm{TEST}}\right)}{\sigma \mathrm{TE}P_{\mathrm{TEST}}}$$

where TEP_CONDITION_ represents individual values of TEP_TEST_, TEP_CONT_, TEP_PSI_ or TEP_FAC_, and $\mu \mathrm{TE}P_{\mathrm{TEST}}$ and $\sigma \mathrm{TE}P_{\mathrm{TEST}}$ represent the mean and SD of test responses, respectively. This standardization allowed direct comparison of modulation across conditions despite the absence of M_max_ normalization.

The degree of D_1_ and D_2_ inhibition, and that of HF, was examined by the mean of the conditioned response at each ISI (i.e., H_PSI_ or H_FAC_ and TEP_PSI_ or TEP_FAC_) expressed as a percentage of the mean of the unconditioned response (i.e., H_TEST_ or TEP_TEST_), giving the following ratios: H_PSI_/H_TEST_ and TEP_PSI_/TEP_TEST_ for D_1_ and D_2_ inhibition, and H_FAC_/H_TEST_ and TEP_FAC_/TEP_TEST_ for HF.

### 2.9 Statistical analysis

Data in text and tables are expressed as the mean ± SD and as individual values, and data in figures are expressed as the mean ± SD. For tSCS‐evoked responses of the other muscles, *z*‐scores are also reported; however, mean and SD values are additionally presented to facilitate interpretation. In text and figures, the first ISIs correspond to the ones used for the H reflex, while the ISIs in parentheses correspond to those used for TEP. The normality of data distribution was checked using the Shapiro–Wilk test. For values that did not follow a normal distribution, a logarithmic transformation was carried out (for M_at_H/M_max_ of both experiments). A significance level of *p* < 0.05 was accepted for all analyses.

ANOVAs and Student's paired *t*‐tests were performed using Statistica software (v.14.0.1.25; Statsoft, Tulsa, OK, USA). When ANOVAs were performed, if a significant main or interaction effect was observed, Tukey's *post hoc* test was used. Effect sizes were calculated and reported as partial η squared (η_p_^2^), with small, moderate and large effects considered for η_p_^2^ ≥ 0.01, η_p_^2^ ≥ 0.06 and η_p_^2^ ≥ 0.14, respectively, and as Cohen's *d*, with small, moderate and large effects considered for *d* ≥ 0.2, *d* ≥ 0.5 and *d* ≥ 0.8, respectively.

R statistical software (v.4.5; R Core Team, 2025) was used to perform both the linear mixed models (LMMs), using the *nmle* package, and the repeated‐measures correlations, using the *rmcorr* R package (Bakdash & Marusich, 2017). For LMMs, several assumptions were tested. The normality and homoscedasticity of the residuals were checked by visually inspecting residual and Q–Q plots. Linearity was evaluated by inspecting plots of observed versus fitted values. The independence of residuals was checked using autocorrelation function plots. Additionally, model convergence diagnostics were reviewed to ensure that no variance components were estimated as zero, confirming that the random effect structure was free of singularity. When a significant main or interaction effect was observed, *post hoc* contrasts comparing each condition with the TEST baseline were performed, using estimated marginal means, with Holm correction for multiple comparisons, via the *emmeans* package. Additionally, *F*‐values and degrees of freedom were estimated using the Satterthwaite approximation. Effect sizes (η_p_^2^) for fixed effects were computed using the *effectsize* package to aid in the interpretation of the magnitude of the observed effects.

For both experiments, to test the presence of the post‐activation depression mechanism for SOL, a two‐way ANOVA was performed [response (H vs. TEP) × condition (response 1 vs. response 2)]. Moreover, Student's paired *t*‐tests were used to compare the degree of post‐activation depression between the two stimulation types by comparing H_2_/H_1_ and TEP_2_/TEP_1_. The TEP_1_ and TEP_2_ of the other tested muscles were also compared by means of Student's paired *t*‐tests. To ensure consistent stimulation conditions, the M_at_H_TEST_/M_max_ was compared between the two test series for PTNS using Student's paired *t*‐test. For tSCS, the TEP of the L SOL was used as a reference, because it is not supposed to be influenced by the conditioning stimulation (Pierrot‐Deseilligny & Burke, 2005). Therefore, the L SOL TEP_TEST_ was compared with TEP_CONT_, also using Student's paired *t*‐test.

For Experiment 1, a linear mixed‐effects model with response (H vs. TEP) × condition [TEST, CONT, PSI 1 (11), PSI 5 (15), PSI 10 (20), PSI 15 (25), PSI 20 (30) and PSI 25 (35) ms] as fixed effects and random intercepts for participants was fitted to assess D_1_ inhibition. It should be noted that D_1_ and D_2_ inhibition were analysed separately, owing to the differences in sample size (*n* = 20 for D_1_ and *n *= 10 for D_2_). The same LMM structure [response (H vs. TEP) × condition (TEST, CONT, PSI 50 (60), PSI 100 (110) and PSI 200 (210) ms)] was applied for D_2_ inhibition.

Likewise, for Experiment 2, a LMM with response (H vs. TEP) × condition [TEST, CONT, FAC‐1 (9), FAC‐2 (8), FAC‐4 (6), FAC‐6 (4), FAC‐8 (2) and FAC‐10 (1) ms] as fixed effects and random intercepts for participants was used in order to evaluate facilitation across the two types of stimulation.

Subsequently, for each experiment, repeated‐measures correlations were used to relate the degree of inhibition (H_PSI_/H_TEST_ and TEP_PSI_/TEP_TEST_) or facilitation (H_FAC_/H_TEST_ and TEP_FAC_/TEP_TEST_) between the two responses, across ISIs within each participant.

For tSCS‐evoked responses recorded on each of the other tested muscles, statistical analysis was performed on *z*‐scores calculated relative to the TEST condition for each participant and muscle. These *z*‐scores were analysed using LMMs with condition [(TEST, CONT, PSI 11, PSI 15, PSI 20, PSI 25, PSI 30 and PSI 35 ms) for D_1_, (TEST, CONT, PSI 60, PSI 110, PSI 210 ms) for D_2_ and (TEST, CONT, FAC 9, FAC 8, FAC 6, FAC 4, FAC 2 and FAC 1 ms) for HF] as a fixed effect and participant as a random intercept.

Finally, for both experiments, an additional analysis was performed using repeated‐measures correlations to compare the modulations observed for TEP of SOL muscle with those observed for TEP of the conditioning muscle. For this purpose, SOL TEP_PSI_/ TEP_TEST_ evoked at each ISI were compared with TA TEP_PSI_/TEP_TEST_ for D_1_ and D_2_, and SOL TEP_FAC_/TEP_TEST_ evoked at each ISI were compared with VL TEP_FAC_/TEP_TEST_ for HF.

## 3 RESULTS

### 3.1 Common results between the two experiments

#### 3.1.1 Post‐activation depression

For Experiment 1, the two‐way ANOVA [response (H vs. TEP) × condition (response 1 vs. response 2)] performed on SOL muscle revealed only an effect of condition (*F*_1,19_ = 166.85, *p* < 0.001, η_p_^2^ = 0.90). Tukey's *post hoc* test showed that H_1_/M_max_ (0.46 ± 0.17) and TEP_1_/M_max_ (0.50 ± 0.12) were higher than H_2_/M_max_ (0.23 ± 0.15) and TEP_2_/M_max_ (0.27 ± 0.14) (*p* < 0.001). In the same way for Experiment 2, only an effect of condition was observed (*F*_1,19_ = 101.47, *p* < 0.001, η_p_^2^ = 0.84), with the first response (0.49 ± 0.25 and 0.50 ± 0.22, for H_1_/M_max_ and TEP_1_/M_max_, respectively) being higher than the second one (0.25 ± 0.16 and 0.23 ± 0.14, for H_2_/M_max_ and TEP_2_/M_max_, respectively) (*p* < 0.001). Moreover, the comparison between H_2_/H_1_ (0.47 ± 0.18) and TEP_2_/TEP_1_ (0.52 ± 0.20) revealed that the degree of inhibition was not significantly different between the two types of stimulation for Experiment 1 (*p* = 0.31, *d* = 0.23) and Experiment 2 (0.52 ± 0.20 and 0.46 ± 0.17, for H_2_/H_1_ and TEP_2_/ TEP_1_, respectively, *p* = 0.26, *d* = 0.29). For tSCS‐evoked responses of the other recorded muscles, except for L and R VL muscles, post‐activation depression was present, because TEP_1_ was significantly higher than TEP_2_ (Table 1).

**TABLE 1:** Transcutaneous spinal cord stimulation‐evoked responses recorded using paired‐pulse stimulations to evaluate the post‐activation depression phenomenon on muscles of the lower limb.

	Experiment 1			Experiment 2		
Muscle	TEP_1_	TEP_2_	*P‐*values/ES	TEP_1_	TEP_2_	*p*‐Value/ES
**GL**	1.74 ± 0.67	0.98 ± 0.60***	**<0.001/0.99**	1.77 ± 0.89	0.92 ± 0.51***	**<0.001/1.26**
**GM**	2.26 ± 2.28	1.37 ± 1.52***	**<0.001/1.04**	1.69 ± 1.11	0.83 ± 0.56***	**<0.001/1.00**
**TA**	0.61 ± 0.36	0.52 ± 0.45**	**0.006/0.70**	0.53 ± 0.28	0.37 ± 0.26**	**0.01/0.73**
**VL**	0.60 ± 0.76	0.53 ± 0.60	0.12/0.42	0.31 ± 0.17	0.29 ± 0.18	0.18/0.34
**L SOL**	6.47 ± 3.44	4.69 ± 3.35***	**<0.001/1.27**	5.91 ± 4.15	4.05 ± 4.07***	**<0.001/1.08**
**L VL**	0.36 ± 0.19	0.35 ± 0.23	0.82/0.06	0.28 ± 0.25	0.24 ± 0.22	0.23/0.32

*Note*: Values are the means ± SD and are expressed in millivolts [*n* = 20 for Experiment 1 (except for GM *n* = 18, VL *n* = 19 and L VL *n* = 16) and *n* = 16 for Experiment 2 (except for VL *n* = 15 and L VL *n* = 13)]. Student's paired *t*‐test; **p < 0.01 and ***p < 0.001 significantly different from TEP_1_. Significant *p*‐values are shown in bold. Abbreviations: ES, effect size (η_p_^2^); GL, gastrocnemius lateralis; GM, gastrocnemius medialis; L SOL, left soleus muscle; L VL, left vastus lateralis muscle; TA, tibialis anterior; TEP, transspinal evoked potential; VL, vastus lateralis.

#### 3.1.2 Constancy of stimulation conditions

Student's paired *t*‐test showed that M_at_H_TEST_/M_max_ was not significantly different from M_at_H_CONT_/M_max_ for both experiments (for all, *p* > 0.05). Additionally, no difference was observed between TEP_TEST_ and TEP_CONT_ of left SOL for D_1_, D_2_ and HF (for all, *p* > 0.05; Table 2). These results highlight the steadiness of stimulation conditions throughout each experimental session.

**TABLE 2:** Values of electrophysiological responses permitting control of the constancy of stimulation conditions throughout the two experimental sessions.

	M_at_H_TEST_/M_max_	M_at_H_CONT_/M_max_	*p*‐Value/ES	L SOL TEP_TEST_	L SOL TEP_CONT_	*p*‐Value/ES
Experiment 1. D_1_	0.09 ± 0.07	0.09 ± 0.08	0.07/0.43	6.76 ± 3.54	6.35 ± 3.74	0.53/0.14
Experiment 1. D_2_	0.09 ± 0.09	0.09 ± 0.09	0.39/0.28	6.33 ± 3.73	6.26 ± 3.79	0.62/0.17
Experiment 2. HF	0.07 ± 0.05	0.07 ± 0.06	0.41/0.21	5.69 ± 4.35	5.85 ± 4.35	0.16/0.35

*Note*: Values are means ± SD (*n* = 20 for D_1_, *n* = 10 for D_2_ and *n* = 16 for HF). Student's paired *t*‐test. Abbreviations: ES, effect size (η_p_^2^); HF, heteronymous facilitation; L SOL, left soleus muscle; L SOL TEP_TEST_ and TEP_CONT_, unconditioned TEP evoked on the left SOL muscle (raw values are expressed in millivolts); M_at_H, M‐wave associated with H reflex of SOL; TEP, transspinal evoked potential.

### 3.2 Experiment 1: D_1_ and D_2_ inhibition

#### 3.2.1 Modulation of H reflex and TEP of the soleus muscle

**FIGURE 2:**
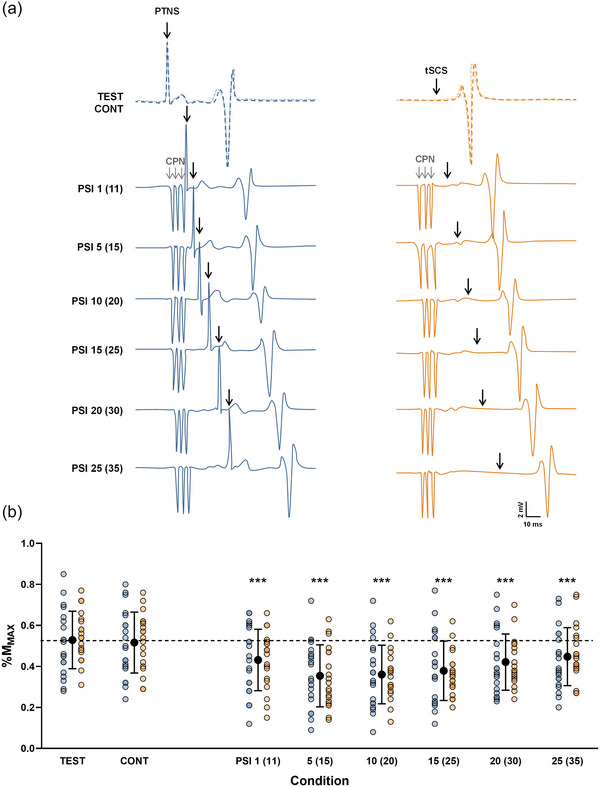
Evolution of SOL D_1_ inhibition. (a) Recordings from a representative participant of unconditioned and conditioned Hoffmann (H) reflex and TEP. The left blue panel corresponds to the evolution of the H reflex and the right orange panel to the evolution of TEP at each ISI. The dotted line represents the amplitude of the test response, while the lighter (more transparent) line represents the amplitude of the control response. Abbreviations: CONT, control response; CPN, common peroneal nerve (conditioning stimulation); ISI, interstimulus interval; PTNS, posterior tibial nerve stimulation; PSI, conditioned response; SOL, soleus muscle; TEP, transspinal evoked potential; TEST, test response; tSCS, transcutaneous spinal cord stimulation. (b) Test, control and conditioned responses evoked at each ISI. Black symbols represent the mean ± SD for each condition (pooled data of H reflex and TEP). Individual values for H_TEST_/M_max_, H_CONT_/M_max_ and H_PSI_/M_max_ evoked at each ISI are shown in blue; TEP_TEST_/M_max_, TEP_CONT_/M_max_ and TEP_PSI_/M_max_ in orange. The dashed line indicates the mean test response value (pooled data of H reflex and TEP). Linear mixed model (*n* = 20). ***p < 0.001, significantly different from TEST. Although no interaction effect was observed, evolution of each type of response is presented separately for more clarity.

As depicted in Figure 2, for D_1_ inhibition, the LMM revealed a significant main effect of condition (*F*_7,180.02_ = 28.65, *p* < 0.001, η_p_^2^ = 0.42). No significant response (*F*_1,280.04_ = 0.02, *P* = 0.89, η_p_^2^ = 0.002) or response × condition interaction effects (*F*_7,280.02_ = 0.93, *p* = 0.49, η_p_^2^ = 0.02) were observed. *Post hoc* contrast analysis indicated that the control series was not significantly different from the test one, while a significant reduction in responses for all tested ISIs compared with the TEST conditions was observed (*p* < 0.001).

**FIGURE 3:**
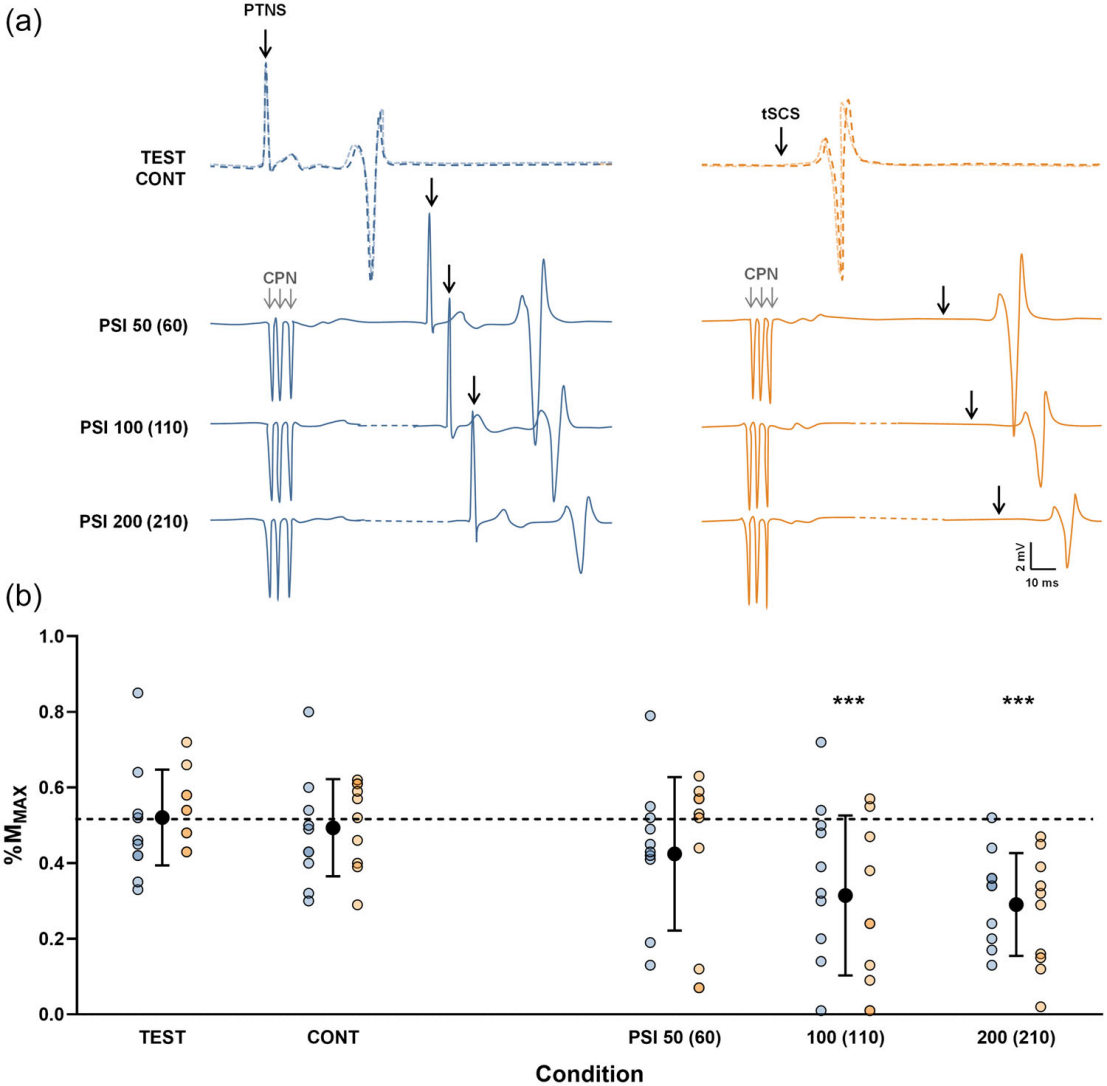
Evolution of SOL D_2_ inhibition. (a) Recordings from a representative participant of unconditioned and conditioned Hoffmann (H) reflex and TEP. The left blue panel corresponds to the evolution of H reflex and the right orange panel to the evolution of TEP at each interstimulus interval. The dotted line represents the amplitude of the test response, while the lighter (more transparent) line represents the amplitude of the control response. Abbreviations: CONT, control response; CPN, common peroneal nerve (conditioning stimulation); PTNS, posterior tibial nerve stimulation; PSI, conditioned response; SOL, soleus muscle; TEP, transspinal evoked potential; TEST, test response; tSCS, transcutaneous spinal cord stimulation. (b) Test, control and conditioned responses evoked at each ISI. Individual values for H_TEST_/M_max_, H_CONT_/M_max_ and H_PSI_/M_max_ evoked at each ISI are shown in blue; TEP_TEST_/M_max_, TEP_CONT_/M_max_ and TEP_PSI_/M_max_ in orange. Black symbols represent the mean ± SD for each condition (pooled data of H reflex and TEP). The dashed line indicates the mean test response value (pooled data of H reflex and TEP). Linear mixed model (*n* = 10). ***p < 0.001 versus TEST. Although, no interaction effect was observed, evolution of each type of response is presented separately for more clarity.

Regarding D_2_ inhibition (Figure 3), the LMM revealed a significant main effect of condition (*F*_4,81_ = 11.74, *p* < 0.001, η_p_^2^ = 0.37), whereas no significant main response (*F*_1,4_ = 0.44, *p* = 0.51, η_p_^2^ = 0.005) or response × condition interaction effects (*F*_4,81_ = 0.82, *p* = 0.52, η_p_^2^ = 0.04) were observed. *Post hoc* contrast comparisons, detailed in Figure 3b, showed a decrease of conditioned responses at the 100 (110) and 200 (210) ms ISIs compared with TEST (*p* < 0.001).

**FIGURE 4:**
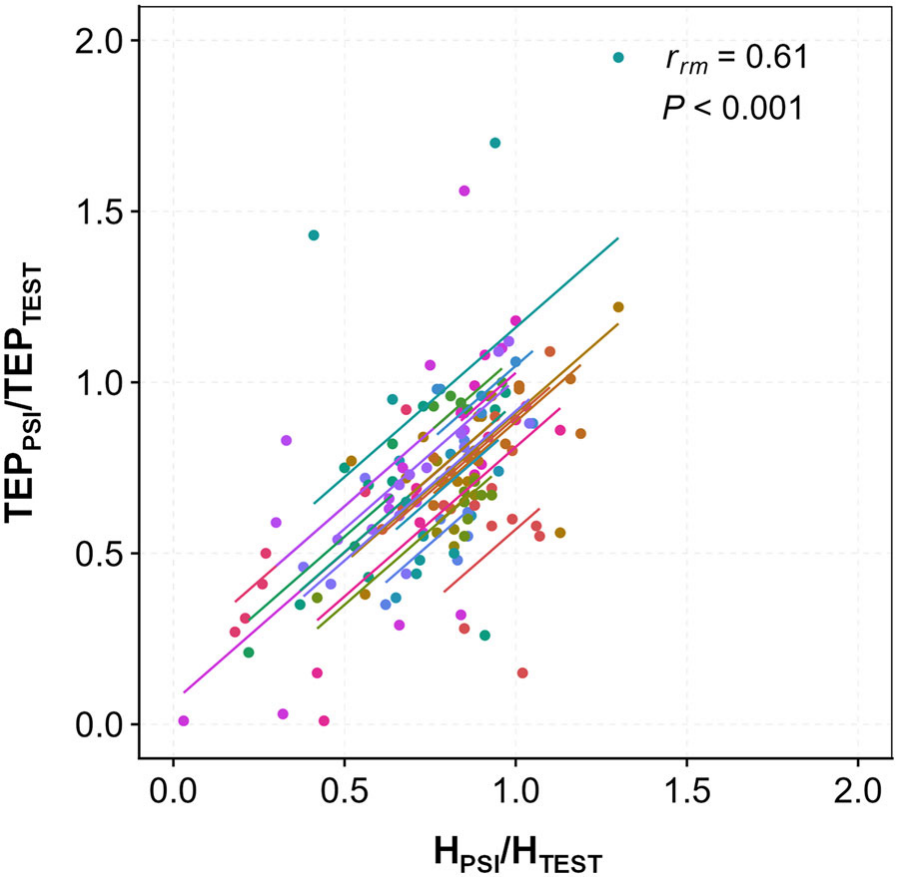
Correlation between the degree of soleus Hoffmann reflex inhibition and the degree of soleus transspinal evoked potential (TEP) inhibition for D_1_ and D_2_ interstimulus intervals (ISIs). Repeated‐measures correlations (*rmcorr*) between H_PSI_/H_TEST_ and TEP_PSI_/TEP_TEST_ are shown across all tested ISIs. A positive linear relationship was obtained. Identically coloured dots represent data from the same participant.

Regarding the degree of D_1_ and D_2_ inhibition between the two types of responses, a positive correlation was observed between H_PSI_/H_TEST_ and TEP_PSI_/TEP_TEST_ across the different tested ISIs [Repeated‐measures correlation *r*_rm_ (128) = 0.61, 95% confidence interval (CI) 0.492, 0.71; *p* < 0.001; Figure 4].

#### 3.2.2 Modulation of TEP evoked in other muscles

**FIGURE 5:**
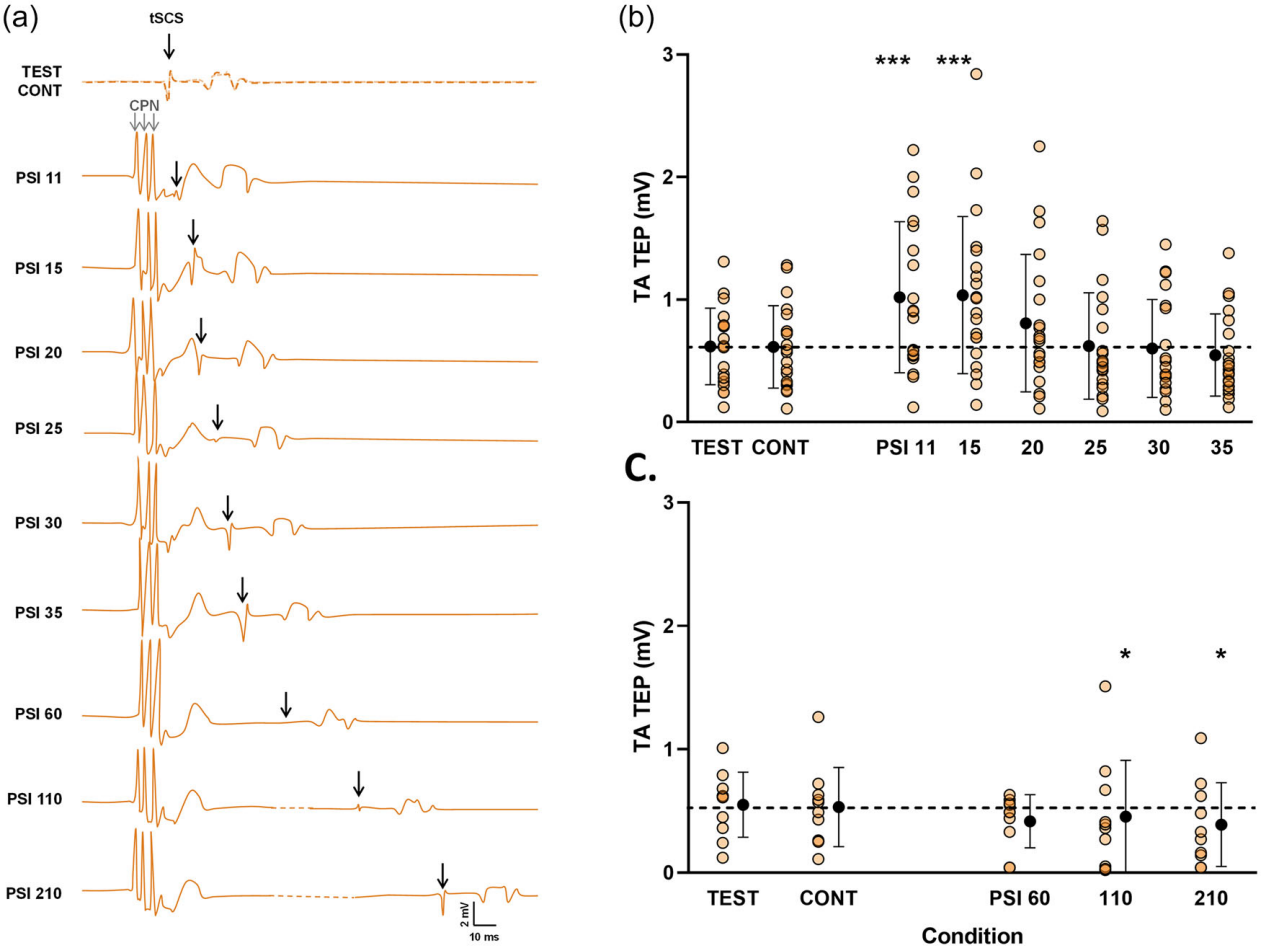
Evolution of TA (conditioning muscle) TEP during D_1_ and D_2_ inhibition. (a) Recordings from a representative participant of unconditioned and conditioned TEP. Each response corresponds to the amplitude of TA TEP evoked when the conditioning stimulation was applied to the common peroneal nerve at different ISIs. The dotted line represents the amplitude of the TA TEP_TEST_, while the lighter (more transparent) line represents the amplitude of TA TEP_CONT_. Abbreviations: CONT, control response; CPN, common peroneal nerve (conditioning stimulation); ISI, interstimulus interval; PSI, conditioned response; TA, tibialis anterior; TEP, transspinal evoked potential; TEST, test response; tSCS, transcutaneous spinal cord stimulation. (b,c) Test, control and conditioned responses evoked at each ISI during D_1_ inhibition (b) and D_2_ inhibition (c). Black symbols represent the mean ± SD for each condition. Individual values for TEP_TEST_, TEP_CONT_ and TEP_PSI_ are in orange. The dashed line indicates the mean test response value. Linear mixed model (*n* = 20 for D_1_ and *n* = 10 for D_2_). *p < 0.05 and ***p < 0.001, significantly different from TEST.

For D_1_ inhibition, LMMs, with condition (TEST, CONT, PSI 11, PSI 15, PSI 20, PSI 25, PSI 30 and PSI 35 ms) as a fixed effect, revealed significant effects for all muscles of the right limb: GL (*F*_7,128.12_ = 15.30, *p* < 0.001, η_p_^2^ = 0.45), GM (*F*_7,115.21_ = 8.69, *P* < 0.001, η_p_^2^ = 0.35), TA (*F*_7,129.04_ = 16.41, *p* < 0.001, η_p_^2^ = 0.47) and VL (*F*_7,124.01_ = 3.73, *P* = 0.001, η_p_^2^ = 0.17). Conversely, no significant effect was observed in the contralateral muscles [for L SOL (*F*_7,126.99_ = 0.97, *P* = 0.46, η_p_^2^ = 0.05) and for L VL (*F*_7,102.01_ = 2.09, *p* = 0.051, η_p_^2^ = 0.12)]. Values and results of *post hoc* contrast analysis are presented in Table 3, and the modulation pattern of the TA conditioning muscle is depicted in Figure 5.

**TABLE 3:** Transspinal evoked potential values of the other recorded muscles during D_1_ inhibition.

Muscle	TEST	CONT	PSI 11	15	20	25	30	35
**GL**								
Mean ± SD	**1.94 ± 0.85**	1.81 ± 0.99	**1.34 ± 0.56**	**1.09 ± 0.53**	**1.22 ± 0.63**	**1.24 ± 0.53**	**1.51 ± 0.70**	**1.73 ± 1.11**
*z*‐Score	**0.000**	−0.264	−0.921***	−1.634***	−1.251***	−1.138***	−0.828***	−0.636**
**GM**								
Mean ± SD	**2.27 ± 2.37**	2.26 ± 2.76	1.67 ± 1.57	**1.32 ± 1.42**	**1.67 ± 2.14**	**1.71 ± 2.08**	1.99 ± 2.22	2.19 ± 2.48
*z*‐Score	**0.000**	−0.015	−0.298	−0.854***	−0.491*	−0.489*	−0.240	−0.132
**TA**								
Mean ± SD	**0.62 ± 0.31**	0.61 ± 0.34	**1.16 ± 0.87**	**1.04 ± 0.67**	0.81 ± 0.56	0.62 ± 0.44	0.60 ± 0.40	0.55 ± 0.34
*z*‐Score	**0.000**	−0.033	0.755***	0.702***	0.343	−0.081	−0.168	−0.255
**VL**								
Mean ± SD	**0.72 ± 0.95**	0.68 ± 0.77	0.68 ± 0.97	**0.68 ± 1.05**	0.62 ± 0.78	0.67 ± 0.96	0.59 ± 0.64	0.68 ± 0.84
*z*‐Score	**0.000**	0.022	−0.141	−0.223*	−0.145	−0.079	−0.036	0.018
**L SOL**								
Mean ± SD	**6.76 ± 3.54**	6.35 ± 3.73	6.79 ± 3.50	6.69 ± 3.62	6.76 ± 3.51	6.54 ± 3.53	6.67 ± 3.74	6.63 ± 3.60
*z*‐Score	**0.000**	−0.012	0.010	0.003	−0.035	−0.045	−0.038	−0.025
**L VL**								
Mean ± SD	**0.70 ± 0.76**	0.57 ± 0.42	0.60 ± 0.65	0.61 ± 0.64	0.58 ± 0.55	0.58 ± 0.54	0.56 ± 0.50	0.58 ± 0.58
*z*‐Score	**0.000**	−0.019	−0.117	−0.104	−0.115	−0.105	−0.142	−0.113

*Note*: Values are means ± SD and are expressed in millivolts (*n* = 20, except for GM *n* = 18, for VL *n* = 19 and for L VL *n* = 16). The *z*‐scores were standardized to test response. Linear mixed model, *p < 0.05, **p < 0.01, ***p < 0.001, significantly different from TEST. The test value and those significantly different from it are shown in bold. Abbreviations: CONT, control response; GL, gastrocnemius lateralis; GM, gastrocnemius medialis; L SOL, left soleus muscle; L VL, left vastus lateralis muscle; PSI, conditioned response; TA, tibialis anterior; TEST, test response; VL, vastus lateralis.

The LMMs conducted to assess D_2_ inhibition showed a significant effect of condition for GL (*F*_4,34.78_ = 4.59, *p* = 0.004, η_p_^2^ = 0.25) and TA (*F*_4,36_ = 4.27, *p* = 0.006, η_p_^2^ = 0.32), whereas no effect was observed for GM (*F*_4,28_ = 2.06, *p* = 0.114, η_p_^2^ = 0.23) and VL (*F*_4,32_ = 1.67, *p* = 0.181, η_p_^2^ = 0.17) of the right limb. For the contralateral limb, statistical analysis revealed no effect for L SOL (*F*_4,32.99_ = 1.05, *p* = 0.39, η_p_^2^ = 0.11). An effect was present for L VL (*F*_4,20_ = 3.62, *p* = 0.022, η_p_^2^ = 0.42), although *post hoc* contrast analysis revealed no statistical difference from the test response for any ISI. Detailed values and results of *post hoc* contrast are provided in Table 4, and the evolution of the TA as the conditioning muscle is illustrated in Figure 5.

**TABLE 4:** Transspinal evoked potential values of the other recorded muscles during D_2_ inhibition.

Muscle	TEST	CONT	PSI 60	110	210
**GL**					
Mean ± SD	**1.77 ± 0.52**	1.44 ± 0.38	**1.30 ± 0.77**	**0.96 ± 0.53**	**0.87 ± 0.44**
*z*‐Score	**0.000**	−0.749	−2.011*	−2.266*	−2.915*
**GM**					
Mean ± SD	**1.29 ± 0.41**	1.22 ± 0.47	1.07 ± 0.60	0.80 ± 0.45	0.68 ± 0.35
*z*‐Score	**0.000**	−0.288	−1.177	−2.837	−3.180
**TA**					
Mean ± SD	**0.55 ± 0.26**	0.53 ± 0.32	0.42 ± 0.21	**0.45 ± 0.45**	**0.39 ± 0.34**
*z*‐Score	**0.000**	−0.112	−0.709	−1.127*	−1.045*
**VL**					
Mean ± SD	**0.42 ± 0.53**	0.47 ± 0.59	0.41 ± 0.60	0.43 ± 0.61	0.44 ± 0.61
*z*‐Score	**0.000**	0.084	−0.395	−0.304	−0.102
**L SOL**					
Mean ± SD	**6.33 ± 3.73**	6.26 ± 3.79	6.06 ± 3.55	6.41 ± 3.66	6.35 ± 3.74
*z*‐Score	**0.000**	−0.006	−0.017	0.033	−0.031
**L VL**					
Mean ± SD	**0.41 ± 0.23**	0.46 ± 0.20	0.41 ± 0.23	0.52 ± 0.25	0.50 ± 0.22
*z*‐Score	**0.000**	0.260	−0.132	0.522	0.459

*Note*: Values are means ± SD and are expressed in millivolts (*n* = 10, except for L VL *n* = 9). The z‐scores were standardized to test response. Linear mixed model, *p < 0.05, **p < 0.01, ***p < 0.001, significantly different from TEST. The test value and those significantly different from it are shown in bold. Abbreviations: CONT, control response; GL, gastrocnemius lateralis; GM, gastrocnemius medialis; L SOL, left soleus muscle; L VL, left vastus lateralis muscle;PSI, conditioned response; TA, tibialis anterior; TEST, test response; VL, vastus lateralis.

### 3.3 Experiment 2: Heteronymous facilitation

#### 3.3.1 Modulation of H reflex and TEP of the soleus muscle

**FIGURE 6:**
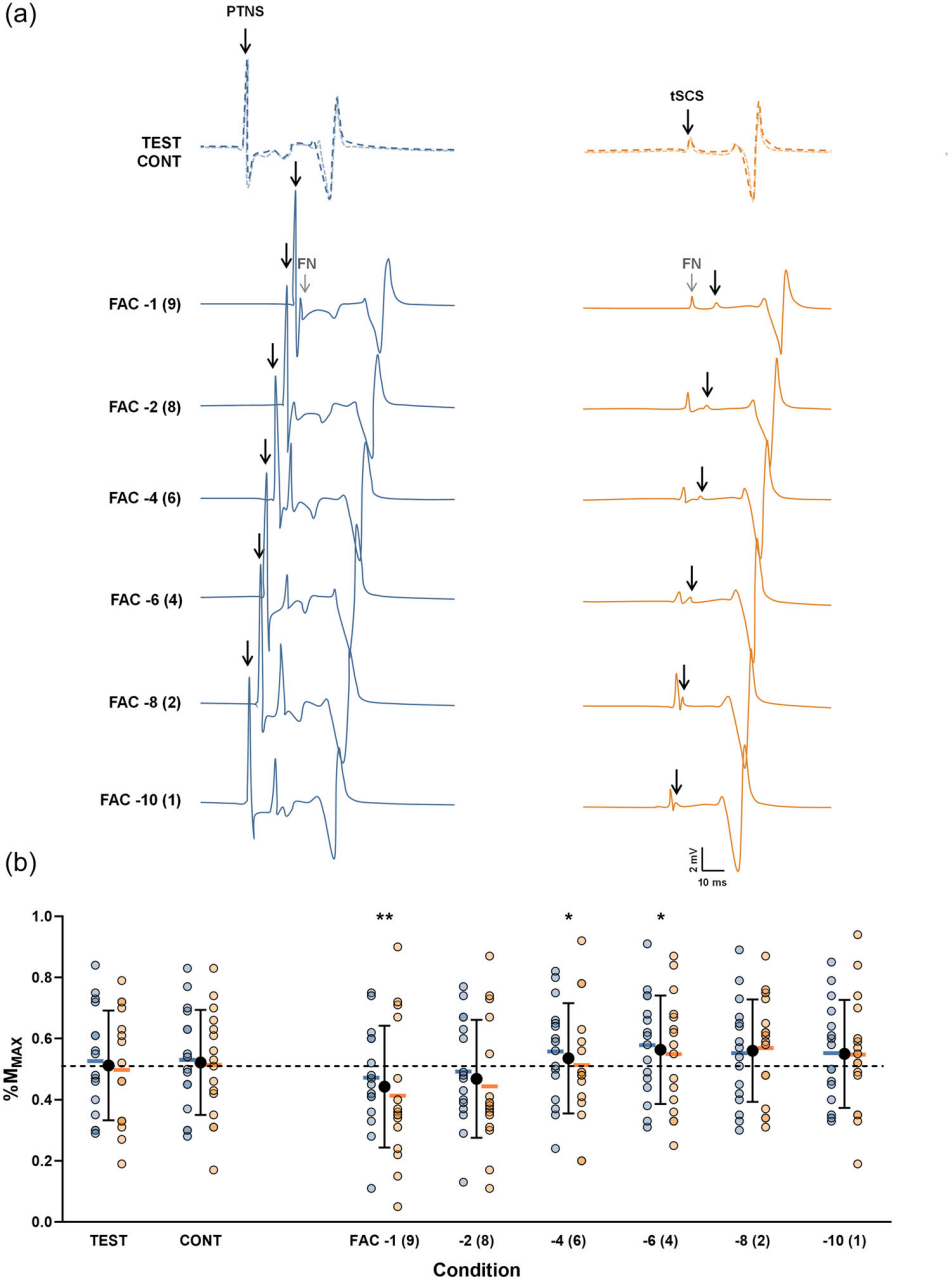
Evolution of SOL heteronymous facilitation. (a) Recordings from a representative participant of unconditioned and conditioned Hoffmann (H) reflex and TEP. The left blue panel corresponds to the evolution of H reflex and the right orange panel to the evolution of TEP at each ISI. The dotted line represents the amplitude of the test response, while the lighter (more transparent) line represents the amplitude of the control response. Abbreviations: CONT, control response; FAC, conditioned response, FN, femoral nerve (conditioning stimulation); ISI, interstimulus interval; PTNS, posterior tibial nerve stimulation; SOL, soleus; TEP, transspinal evoked potential; TEST, test response; tSCS, transcutaneous spinal cord stimulation. (b) Test, control and conditioned responses evoked at each ISI. Individual values for H_TEST_/M_max_, H_CONT_/M_max_ and H_FAC_/M_max_ evoked at each ISI are shown in blue; TEP_TEST_/M_max_, TEP_CONT_/M_max_ and TEP_FAC_/M_max_ in orange. For each condition, the mean is represented by a horizontal line to highlight the reflex effect. Black symbols represent the mean ± SD for each condition. The dashed line indicates the mean test response value. Linear mixed model (*n* = 16). ***p < 0.001 versus TEST. Although no interaction effect was observed, the evolution of each type of response is presented separately for clarity.

As illustrated in Figure 6, facilitation was not observed consistently across all tested ISIs. The LMM revealed a significant main effect of response (*F*_1,225_ = 8.00, *p* = 0.005, η_p_^2^ = 0.03) and a significant main effect of condition (*F*_7,225_ = 10.98, *p* < 0.001, η_p_^2^ = 0.25). No significant response × condition interaction was found (*F*_7,225_ = 0.88, *p* = 0.52, η_p_^2^ = 0.03). *Post hoc* contrast analysis revealed a significant difference between H reflex and TEP (*p* < 0.01, *d* = 0.26), with the H reflex being significantly higher than the TEP across all conditions (*p* < 0.01). *Post hoc* contrast performed for the effect of condition is detailed in Figure 6b and showed that FAC‐1 (9) was significantly lower than TEST (*p* = 0.002) and that FAC‐4 (6) and FAC‐6 (4) were significantly higher than TEST (*p* = 0.03 and *p* = 0.04, respectively).

**FIGURE 7:**
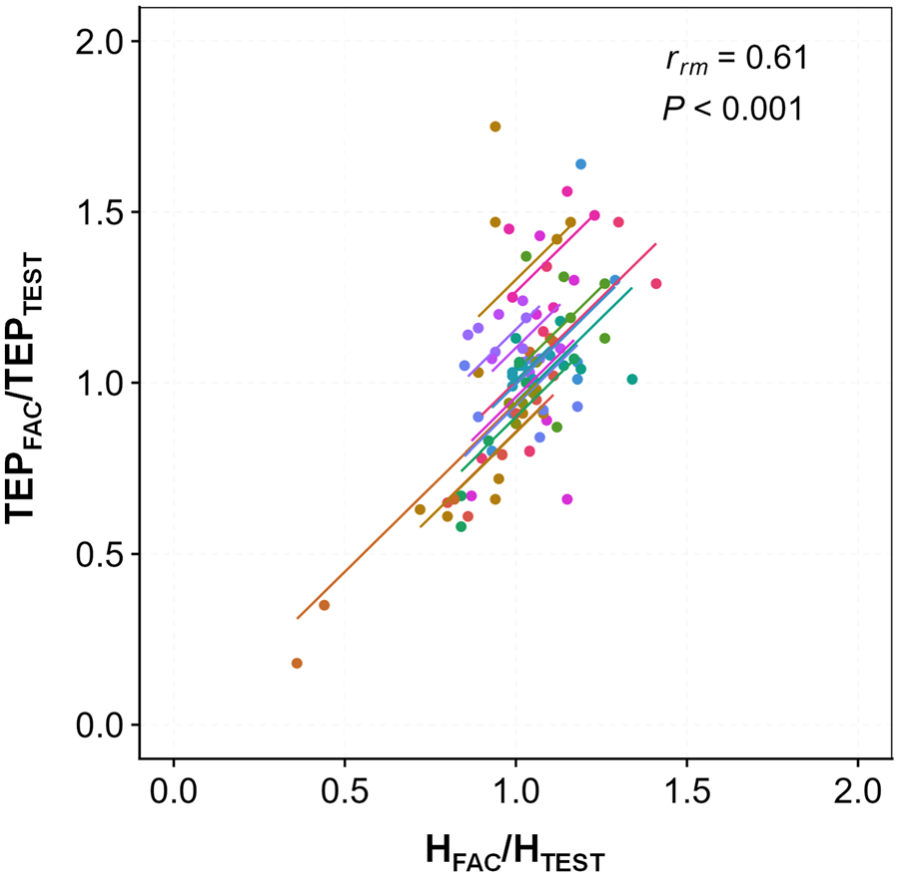
Correlation between the degree of soleus Hoffmann (H) reflex facilitation and the degree of soleus transspinal evoked potential (TEP) facilitation across interstimulus intervals. Repeated‐measures correlations (*rmcorr*) are shown between H_FAC_/H_TEST_ and TEP_FAC_/TEP_TEST_. A positive linear relationship was obtained. Identically coloured dots represent data from the same participant.

In Exeperiment 2 (as in Experiment 1), a positive correlation was found between H_FAC_/H_TEST_ and TEP_FAC_/TEP_TEST_ across all tested ISIs [*r*_rm_ (79) = 0.61, 95% CI 0.451, 0.73; *p *< 0.001; Figure 7].

#### 3.3.2 Modulation of TEP evoked on other muscles

**FIGURE 8:**
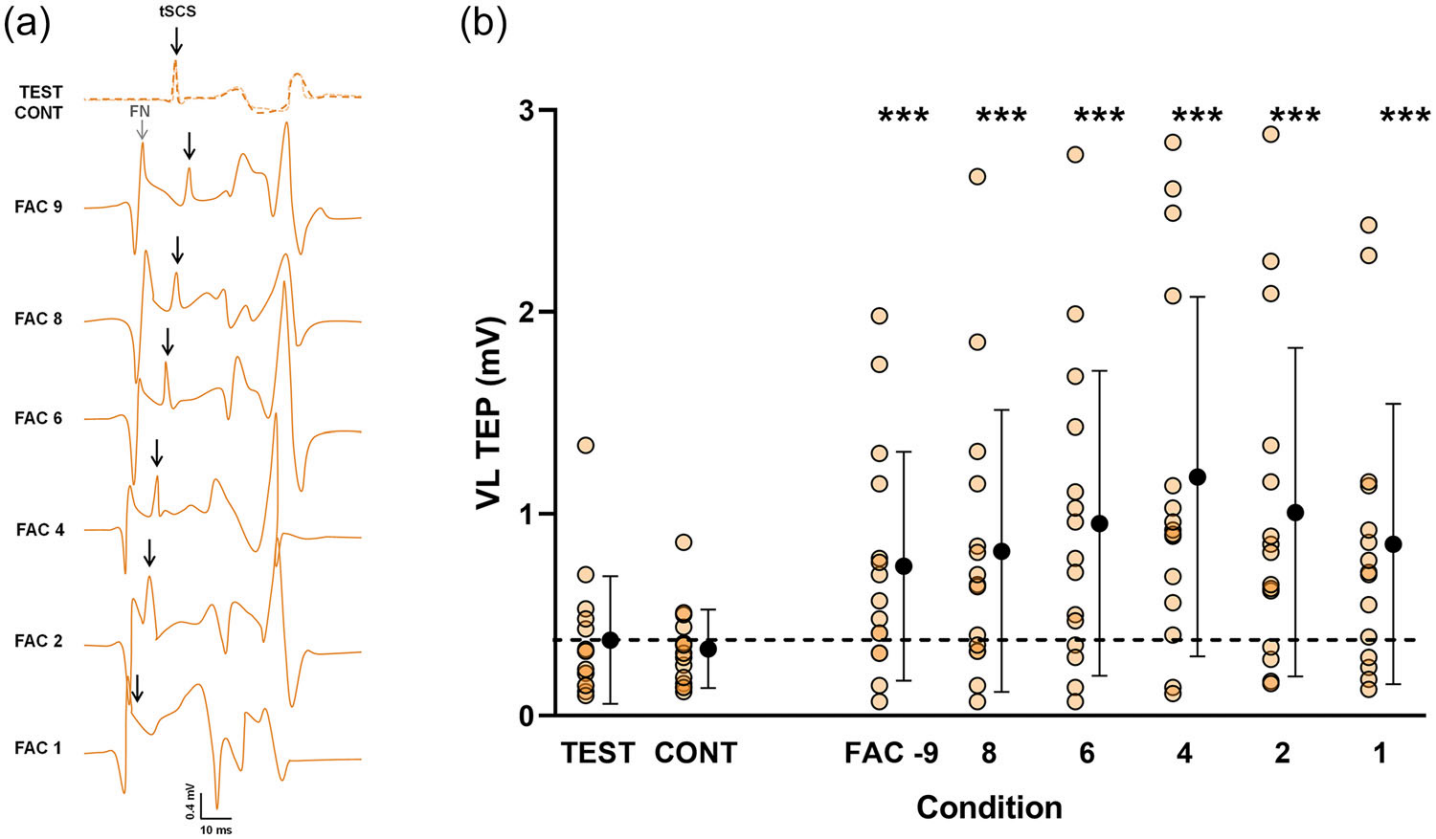
Evolution of VL (conditioning muscle) TEP during heteronymous facilitation. (a) Recordings from a representative participant of unconditioned and conditioned TEP. Each response corresponds to the amplitude of VL TEP when the conditioning stimulation was applied to the common peroneal nerve at different ISIs. The dotted line represents the amplitude of the VL TEP_TEST_, while the lighter (more transparent) line represents the amplitude of VL TEP_CONT_. (b) Test, control and conditioned responses evoked at each ISI during heteronymous facilitation. Individual values for TEP_EST_, TEP_CONT_ and TEP_FAC_ are shown in orange. Black symbols represent the mean ± SD for each condition. The dashed line indicates the mean test response value. Linear mixed model (*n* = 15). ***p < 0.001 versus TEST. Abbreviations: CONT, control response; FAC, conditioned response; FN, femoral nerve (conditioning stimulation); ISI, interstimulus interval; TEST, test response; tSCS, transcutaneous spinal cord stimulation; TEP, transspinal evoked potential; VL, vastus lateralis.

The LMMs revealed significant effects for GL (*F*_7,101.98_ = 5.84, *p* < 0.001, η_p_^2^ = 0.29), GM (*F*_7,104.007_ = 6.30, *p* < 0.001, η_p_^2^ = 0.30), TA (*F*_7,100.23_ = 6.34, *p* < 0.001, η_p_^2^ = 0.31) and VL (*F*_7,98_ = 11.04, *p* < 0.001, η_p_^2^ = 0.44) muscles of the right limb. In the contralateral limb, no significant effect was found for L SOL (*F*_7,97.99_ = 0.54, *p* = 0.80, η_p_^2^ = 0.04). A significant effect of condition was observed for L VL (*F*_7,79.01_ = 2.53, *p* = 0.021, η_p_^2^ = 0.18); however, *post hoc* contrast analysis revealed no significant difference compared with TEP_TEST_. Detailed values and results of *post hoc* contrast comparisons are presented in Table 5. The evolution of the VL as the conditioning muscle is illustrated in Figure 8.

**TABLE 5:** Transspinal evoked potential values of the other recording muscles during heteronymous facilitation.

Muscle	TEST	CONT	FAC 9	8	6	4	2	1
**GL**								
Mean ± SD	**1.76 ± 0.93**	1.79 ± 0.71	**1.30 ± 0.64**	**1.38 ± 0.59**	1.59 ± 0.60	1.66 ± 0.74	1.77 ± 0.86	1.79 ± 0.71
*z*‐Score	**0.000**	0.030	−0.539**	−0.446**	−0.119	−0.095	0.061	0.097
**GM**								
Mean ± SD	**1.76 ± 1.18**	1.78 ± 1.27	**1.44 ± 1.12**	1.63 ± 1.31	1.82 ± 1.37	1.90 ± 1.28	**2.04 ± 1.16**	2.02 ± 1.19
*z*‐Score	**0.000**	0.020	−0.277*	−0.105	0.074	0.159	0.295*	0.199
**TA**								
Mean ± SD	**0.50 ± 0.29**	0.51 ± 0.27	**0.43 ± 0.30**	0.46 ± 0.30	0.51 ± 0.31	0.53 ± 0.30	0.55 ± 0.28	0.54 ± 0.28
*z*‐Score	**0.000**	0.048	−0.210*	−0.095	0.055	0.067	0.148	0.134
**VL**								
Mean ± SD	**0.38 ± 0.32**	0.33 ± 0.19	**0.74 ± 0.57**	**0.82 ± 0.70**	**0.95 ± 0.75**	**1.18 ± 0.89**	**1.01 ± 0.81**	**0.85 ± 0.69**
*z*‐Score	**0.000**	−0.039	0.877***	0.948***	1.151***	1.501***	1.292***	1.065***
**L SOL**								
Mean ± SD	**5.69 ± 4.35**	5.85 ± 4.35	6.06 ± 4.14	6.36 ± 4.45	6.43 ± 4.39	6.31 ± 4.32	6.38 ± 4.26	6.16 ± 4.27
*z*‐Score	**0.000**	0.015	0.001	0.032	0.055	0.040	0.038	0.023
**L VL**								
Mean ± SD	**0.43 ± 0.30**	0.36 ± 0.25	0.38 ± 0.30	0.38 ± 0.30	0.39 ± 0.30	0.42 ± 0.29	0.39 ± 0.26	0.37 ± 0.26
*z*‐Score	**0.000**	−0.141	−0.087	−0.117	−0.005	0.012	0.000	0.000

*Note*: Values are means ± SD and are expressed in millivolts (*n* = 16, except for VL *n* = 15 and for L VL *n* = 13). The *z*‐scores were standardized to test response. Linear mixed model, *p < 0.05, **p < 0.01, ***p < 0.001, significantly different from TEST. The test value and those significantly different from it are shown in bold. Abbreviations: CONT, control response; FAC, conditioned response; GL, gastrocnemius lateralis; GM, gastrocnemius medialis; L SOL, left soleus muscle; L VL, left vastus lateralis muscle; TA, tibialis anterior; TEST, test response; VL, vastus lateralis.

### 3.4 Additional results

**FIGURE 9:**
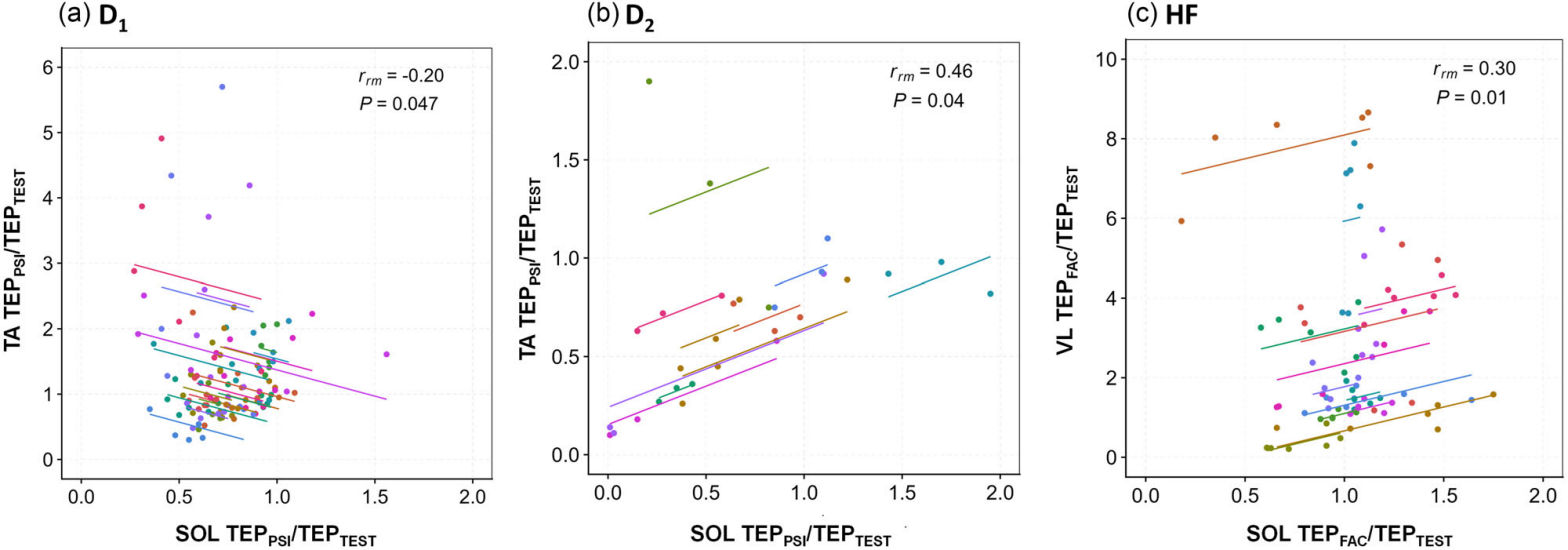
Correlations between the modulation of soleus transspinal evoked potential (SOL TEP) and the modulation of the TEP of the conditioning muscle for D_1_ (a), D_2_ (b) and heteronymous facilitation (c). Repeated‐measures correlations (*rmcorr*) between SOL TEP_PSI_/TEP_TEST_ and TA TEP_PSI_/TEP_TEST_ are shown for D_1_ and D_2_, and between SOL TEP_FAC_/TEP_TEST_ and VL TEP_FAC_/TEP_TEST_ for heteronymous facilitation. Identically coloured dots represent data from the same participant.

Repeated‐measures correlations were performed to compare the modulation of SOL TEP with those of TEP of the conditioning muscle (TA for D_1_ and D_2_ inhibition; VL for HF). For D_1_, a negative correlation was found between SOL and TA TEP_PSI_/TEP_TEST_ [*r*_rm_ (99) = −0.20, 95% CI −0.379, −0.003; *p *= 0.047; Figure 9a], suggesting that the more the TA TEP is facilitated, the more the TEP of SOL is inhibited. Conversely, for D_2_, a positive correlation was observed [*r*_rm_ (19) = 0.46, 95% CI 0.03, 0.742; *p *= 0.04; Figure 9b]. Finally, for HF, a positive correlation was observed between SOL and VL TEP_FAC_/TEP_TEST_ [*r*_rm_ (79) = 0.30, 95% CI 0.069, 0.497; *p *= 0.01; Figure 9c], suggesting that SOL TEPs are most facilitated when a facilitation is also observed for the VL.

## 4 DISCUSSION

The purpose of the present study was to investigate the influence of inhibitory and facilitatory circuits on TEPs elicited by tSCS and on the H reflex evoked by PTNS. The main results showed that both responses of the soleus muscle were sensitive to D_1_ and D_2_ presynaptic inhibition and that the amount of inhibition was similar across the tested ISIs for the two responses. Regarding HF, although this phenomenon is more difficult to elicit and shows greater variability, the H reflex and TEP appeared to follow a similar time course across the tested ISIs. In the present experimental conditions, providing the possibility to examine the concomitant behaviour of multiple muscles, the responses of the synergistic and antagonistic muscles were modulated differently according to the ISI that was used, whereas contralateral muscles did not seem to be impacted by the conditioning stimulations.

### 4.1 Methodological considerations

In this study, tSCS was applied to obtain SOL TEP resulting mainly from activation of Ia afferents. It is well known that some parameters, such as the electrode placement or the intensity of stimulation (Krenn et al., 2015; Roy et al., 2012), can influence the nature of TEP, which results from depolarization of both Ia afferents and motor axons. The cathode in the present study was placed at the L1–L2 level of the spinal cord, because at this placement: (1) the intensity threshold to evoke SOL TEP is lower than at higher levels of electrode location (Sayenko et al., 2015); (2) sensory fibres of SOL are preferentially activated; and (3) motor axons of VL are mainly activated, minimizing the heteronymous contribution to the SOL motoneuron pool (Roy et al., 2012). To match the test responses, the H reflex and TEP amplitudes were fixed at 80% H_max_. This target amplitude was chosen because during PTNS it permits the recording of a submaximal M‐wave preceding the H reflex, providing the possibility to control the constancy of the stimulus intensity (Theodosiadou et al., 2023) and because the intensity used to reach this amplitude is below the tolerable pain threshold for tSCS. In the present study, to make sure that tSCS preferentially activated Ia afferents of SOL and motor axons of VL, the post‐activation depression phenomenon was tested at the beginning of each experimental session. A decrease of the second TEP was observed for SOL, and the second VL TEP was not different from the first one, indicating preferential activation of motor axons for VL. Moreover, the degree of post‐activation depression was similar between the SOL H reflex and TEP, suggesting that the same proportion of Ia afferents was activated by the two types of stimulation. In these conditions, the comparison between tSCS and PTNS was possible. Finally, as in previous studies comparing the two types of stimulation (Andrews et al., 2015), each ISI was adjusted for tSCS owing to the proximity of the stimulation site to the spinal cord and the shorter response latency of TEP compared with the H reflex (10 ms difference) (Kitano & Koceja, 2009).

### 4.2 Presynaptic inhibition of SOL responses

When PTNS is preceded by stimulation of the common peroneal nerve, a decrease of the SOL H reflex is observed in comparison to unconditioned PTNS. This decrease is attributed to the activation of PAD interneurons owing to the solicitation of Ia afferents of the antagonist muscle (TA muscle). It has been suggested that the mechanisms involved in D_1_ and D_2_ presynaptic inhibition seem to imply modulations of the Ia–α‐motoneuron synapse through GABA_A_ receptors (Rudomin & Schmidt, 1999). The activation of GABA_A_ receptors on Ia terminals increases Cl^−^ ion efflux and produces depolarization of the afferent synapse. This leads to a reduction in the amplitude of the propagated action potential, thus reducing Ca^2+^ influx and, consequently, the amount of neurotransmitter release (Pierrot‐Deseilligny & Burke, 2005; Rudomin & Schmidt, 1999). However, recent studies have challenged the role of PAD and GABA_A_ receptors in producing presynaptic inhibition in the Ia afferent terminal (Hari et al., 2022; Metz et al., 2023). It has been shown that instead of presynaptic inhibition, the activation of PAD interneurons by GABA_A_ receptors has a facilitatory role on Ia afferent conduction. It was suggested that the decrease of the H reflex caused by solicitation of antagonist afferents could be attributable to a direct long‐lasting inhibition of the test motoneurons and/or from post‐activation depression of Ia afferent transmission (Metz et al., 2023). Independently of the exact mechanism inducing D_1_ and D_2_ inhibition, the exploration of presynaptic inhibition in this study permitted us to obtain a further comparison between the H reflex and TEP.

For both responses, inhibition was present across all the tested ISIs for D_1_, because the amplitude of the conditioned response was consistently lower than the test response. Concerning D_2_ inhibition, a decrease of the H reflex and TEP was observed at the 100 (110) and 200 (210) ms ISIs, following a modulation pattern similar to that previously reported in the literature for the H reflex (Hultborn et al., 1987; Metz et al., 2023; Rudomin & Schmidt, 1999). Notably, the depression observed at the shortest ISI (1 ms for PTNS and 11 ms for tSCS) might be attributable to reciprocal inhibition (i.e., relaxation of the agonist muscle when the antagonist one is contracted), rather than presynaptic inhibition (Pierrot‐Deseilligny & Burke, 2005). Regardless of the exact mechanisms underlying the reduction of H reflex and TEP amplitudes across ISIs, the overall time course of modulation appeared similar for the two types of stimulation during D_1_ and D_2_ inhibition, because no significant difference was found between the two types of responses. Additionally, the positive linear relationship observed between the degree of inhibition of the H reflex and TEP supports the idea that both responses are likely to be modulated by similar underlying mechanisms.

### 4.3 Heteronymous facilitation of SOL responses

Given that presynaptic inhibition can be modulated not only by the excitability of the interneurons but also by a change in the gain of the motoneuron pool, HF is used concomitantly to assess ongoing presynaptic inhibition on heteronymous Ia facilitation of the SOL H reflex. Contrary to the inhibition observed during activation of Ia afferents of the antagonistic muscle, the SOL H reflex can be facilitated by a heteronymous monosynaptic Ia volley from quadriceps (Hultborn et al., 1987; Pierrot‐Deseilligny & Burke, 2005). In the present study, for PTNS, the conditioning stimulation was applied to the femoral nerve after the test stimulation, with ISIs ranging from 1 to 10 ms. As with the inhibition protocol, ISIs for tSCS were adjusted to account for the shorter latency of tSCS‐evoked responses, with conditioning stimulation delivered before tSCS. The results revealed differences between the two types of reflexes, with H reflex amplitudes being significantly higher than those of the TEP. However, both the H reflex and TEP exhibited a similar pattern of modulation, with facilitation observed at the −4 (6) and −6 (4) ISIs. In contrast, a decrease of conditioned responses evoked at −1 (9) was observed in comparison to the test response. These findings align with previous H reflex studies reporting a greater facilitatory effect at −5 or −6 ms ISIs, with this facilitation diminishing or even disappearing at shorter ISIs (Grosprêtre et al., 2019; Hultborn et al., 1987; Meunier et al., 1996; Pierrot‐Deseilligny & Burke, 2005). Although facilitation was more challenging to assess than presynaptic inhibition, results suggest that both types of responses are influenced in a similar manner by HF, depending on the ISI. This is supported by the positive linear relationship observed between the facilitation levels of the H reflex and TEP.

### 4.4 Behaviour of the other muscles of the lower limb during inhibition

The great interest in using tSCS is to explore the behaviour of several muscles during the conditioning stimulation. During the inhibition experiment, the TA muscle was the conditioning muscle. Results show that TA TEPs were greatly facilitated at the shorter ISIs (11 and 15 ms). By increasing the delay between the common peroneal nerve stimulation and tSCS, this facilitation decreased and then an inhibition was observed for the larger ISIs used to test D_2_ inhibition (110 and 210 ms). Given the latency of the TA TEP (15.2 ± 2.61 ms, present data) and the TA H reflex latency (∼30 ms; Burke et al., 1989), it can be deduced that during common peroneal nerve stimulation, the delay in the activation of TA motoneurons is ∼15 ms. This could explain the facilitation observed on TA TEP when the conditioning stimulation was delivered 11 and 15 ms before tSCS, because both activations must have arrived at approximately the same time at the TA motoneuron pool. For the longer ISIs, the conditioning response should have arrived at the motoneuron level before the tSCS. Hence, the absence of modulation observed at the 20, 25, 30 and 35 ms ISIs might be explained by the after‐hyperpolarization period of TA motoneurons, subsequent to the conditioning stimulation. Finally, at the larger ISIs corresponding to D_2_ inhibition, afferents should have activated motoneurons two distinct times, leading to an inhibition, owing to homonymous post‐activation depression.

Concerning the synergistic muscles, it has already been reported that GL and GM H reflexes are also impacted by inhibitory circuits (Grosprêtre et al., 2019; Ménard et al., 1999; Nielsen & Kagamihara, 1993; Vitry et al., 2021). In the present study, a comparison of the degree of inhibition was not possible between the two types of stimulation for these two muscles, because the amplitudes of H_TEST_ and TEP_TEST_ were not equal (remembering that the intensity of stimulation was adjusted to evoke an H reflex and TEP of the same amplitude on SOL). Although this comparison was not possible, TEPs obtained on these synergistic muscles were also sensitive to presynaptic inhibition, because inhibition was observed for most of the ISIs that were tested in comparison to test responses (except for GM during D_2_ inhibition), consistent with results of previous studies (Grosprêtre et al., 2019; Vitry et al., 2021). Concerning right VL responses, when TA afferents were activated to induce SOL inhibition, a decrease of VL tSCS‐evoked response was observed only at the 15 ms ISI. This result is unexpected, given that TA Ia afferents do not appear to influence quadriceps muscle responses by postsynaptic inhibitory mechanisms (Pierrot‐Deseilligny & Burke, 2005) and that VL tSCS‐evoked responses were primarily efferent in nature, as evidenced by the absence of post‐activation depression for this muscle. However, it can be hypothesized that, despite the absence of post‐activation depression, some afferents might have been activated by tSCS and contributed to the VL tSCS‐evoked response. In this case, the decrease of tSCS‐evoked response observed at the 15 ms ISI might be explained by presynaptic inhibition of the VL TEP, mediated by projections from PAD interneurons—activated by Ia afferents from the TA—onto afferents of the VL (Pierrot‐Deseilligny & Burke, 2005). These modulations, observed for the right limb, were not present in the contralateral muscles (i.e., L SOL and L VL), ensuring that stimulation conditions remained identical throughout the experimental session.

### 4.5 Behaviour of the other muscles of the lower limb during facilitation

During the facilitation experiment, the VL was the conditioning muscle. An increase of VL tSCS‐evoked response in comparison to the test response was observed across all ISIs. This facilitatory effect is probably attributable to the summation of responses from the conditioning stimulation of the femoral nerve and from tSCS. Indeed, as observed in the typical data of Figure 8, the VL waveform is polyphasic, and it is difficult to distinguish between the two responses. In the case of both conditioning stimulation and tSCS activating afferent pathways, an inhibition should have been induced at the shorter ISIs, owing to the after‐hyperpolarization period of VL motoneurons. On the contrary, a facilitation was observed, indicating that the summation of the motor response evoked by tSCS and of the afferent response evoked by the conditioning stimulation should be at the origin of the increased conditioned response.

Concerning the two gastrocnemii, when HF was assessed, an inhibition was observed at the 9 ms ISI for GL, with no facilitation for the other ISIs. Conversely, for GM, a facilitatory effect was present at the 2 ms ISI. For TA, an inhibition was also observed at the 9 ms ISI. This result is consistent with previous observations on the complex effects of femoral nerve stimulation on SOL and TA motoneuron pools. For TA, it was shown that during a 20% maximal voluntary dorsiflexion contraction, stimulation of the femoral nerve induced inhibition of the TA H reflex when the femoral nerve stimulation and the peroneal nerve stimulation (for TA H reflex) were delivered at the same time, most probably attributable to recurrent inhibition, owing to activation of Renshaw cells by quadriceps motoneurons (Meunier et al., 1996). However, their study was conducted during voluntary contraction, whereas in the present study facilitation was assessed in resting conditions, making direct comparison of the results difficult. As for inhibition, no modulation was observed for the contralateral leg.

### 4.6 Interest in using tSCS

When inhibitory or facilitatory circuits at the spinal cord level are evaluated, to the best of our knowledge, the behaviour of the conditioning muscle (i.e., TA for inhibition and quadriceps for facilitation) is never controlled. However, modulation of the responses of the conditioning muscles would lead to changes in the inhibitory or facilitatory effect. The use of tSCS to study spinal circuitry provides the advantage of obtaining responses in the target muscle, synergistic muscles, contralateral muscles and the conditioning muscles. In the present study, linear relationships were obtained between the modulations of TEP of the conditioning muscles and those of the SOL. Indeed, for D_1_, the more the TA TEP was facilitated, the more the SOL TEP was inhibited. Conversely, for D_2_, the SOL inhibition was associated with an inhibition of TA. With regard to facilitation, the more the VL TEP was facilitated, the more facilitation was also observed for SOL muscle. These results suggest that when tSCS is used to study inhibitory and facilitatory circuits, the ISIs that induce modulation of TEP of the conditioning muscle can be used to control the constancy of this modulation throughout the experimental session. Additionally, the evolution of TEPs elicited on the contralateral leg provides another index of the constancy of the stimulation conditions. In these conditions, the use of tSCS extends spinal circuitry exploration to multiple muscles. However, it is important to note that these findings were obtained with specific experimental and stimulation parameters. As such, caution should be exercised when attempting to generalize these observations to other protocols.

## 5 CONCLUSION

Through comparison of the H reflex and TEP regarding presynaptic inhibition, in the present study we not only evaluated the modulations of the two reflexes by inhibitory and facilitatory circuits but also gained new evidence on the interest in using tSCS as a method to explore spinal circuitry. In the tSCS conditions used in this study, the modulations caused by the conditioning stimulations were similar between the H reflex and TEP. The results of this study provide an overview of the impact of inhibitory and facilitatory circuits on muscles of the lower limb, including the behaviour of the conditioning muscle and the impact on the synergistic muscles and on the contralateral leg responses. To test all these muscles using peripheral nerve stimulation, several set‐ups would need to be used. The convenient use of tSCS associated with the sensitivity of tSCS‐evoked responses to presynaptic inhibitory mechanisms pave the way to the possible use of TEP as an index of spinal modulations, although further studies need to be carried out to confirm this.

## AUTHOR CONTRIBUTIONS

Julia Sordet, Maria Papaiordanidou, Ioannis Amiridis, Jean‐Pierre Quenot and Alain Martin designed research. Julia Sordet performed the experiments. Julia Sordet and Nicolas Amiez analysed the data. Julia Sordet, Nicolas Amiez, Maria Papaiordanidou and Alain Martin interpreted the results. All authors participated in the drafting of the manuscript and in revising the work. All authors approved the final version of the manuscript and agree to be accountable for all aspects of the work in ensuring that questions related to the accuracy or integrity of any part of the work are appropriately investigated and resolved. All persons designated as authors qualify for authorship, and all those who qualify for authorship are listed.

## CONFLICT OF INTEREST

The authors declare no conflicts of interest.

## Data Availability

The data that support the findings of this study are available from the corresponding author upon reasonable request.
